# Granulovacuolar Degenerations Appear in Relation to Hippocampal Phosphorylated Tau Accumulation in Various Neurodegenerative Disorders

**DOI:** 10.1371/journal.pone.0026996

**Published:** 2011-11-03

**Authors:** Yuu Yamazaki, Tomoyasu Matsubara, Tetsuya Takahashi, Takashi Kurashige, Eisuke Dohi, Masanori Hiji, Yoshito Nagano, Takemori Yamawaki, Masayasu Matsumoto

**Affiliations:** 1 Department of Clinical Neuroscience and Therapeutics, Hiroshima University Graduate School of Biomedical Sciences, Hiroshima, Japan; 2 Department of General Internal Medicine, Aso Iizuka Hospital, Iizuka, Japan; 3 Department of Neurology, Mifukai Viha-ra Hananosato Hospital, Miyoshi, Japan; Philadelphia VA Medical Center, United States of America

## Abstract

**Background:**

Granulovacuolar degeneration (GVD) is one of the pathological hallmarks of Alzheimer's disease (AD), and it is defined as electron-dense granules within double membrane-bound cytoplasmic vacuoles. Several lines of evidence have suggested that GVDs appear within hippocampal pyramidal neurons in AD when phosphorylated tau begins to aggregate into early-stage neurofibrillary tangles. The aim of this study is to investigate the association of GVDs with phosphorylated tau pathology to determine whether GVDs and phosphorylated tau coexist among different non-AD neurodegenerative disorders.

**Methods:**

An autopsied series of 28 patients with a variety of neurodegenerative disorders and 9 control patients were evaluated. Standard histological stains along with immunohistochemistry using protein markers for GVD and confocal microscopy were utilized.

**Results:**

The number of neurons with GVDs significantly increased with the level of phosphorylated tau accumulation in the hippocampal regions in non-AD neurodegenerative disorders. At the cellular level, diffuse staining for phosphorylated tau was detected in neurons with GVDs.

**Conclusions:**

Our data suggest that GVDs appear in relation to hippocampal phosphorylated tau accumulation in various neurodegenerative disorders, while the presence of phosphorylated tau in GVD-harbouring neurons in non-AD neurodegenerative disorders was indistinguishable from age-related accumulation of phosphorylated tau. Although GVDs in non-AD neurodegenerative disorders have not been studied thoroughly, our results suggest that they are not incidental findings, but rather they appear in relation to phosphorylated tau accumulation, further highlighting the role of GVD in the process of phosphorylated tau accumulation.

## Introduction

Granulovacuolar degeneration (GVD) is one of the pathological hallmarks of Alzheimer's disease (AD) [1] and is defined as electron-dense granules within double membrane-bound cytoplasmic vacuoles, mainly in the hippocampal pyramidal neurons [2].

Attempts to define the molecular composition of GVDs by immunohistochemical methods led to the identification of a large number of possible protein constituents, suggesting a link between GVD and AD-related neurodegeneration. For example, the tau protein found in GVD complexes is antigenically related to that found in paired helical filaments in AD, although antibodies to other forms of tau do not recognize GVDs [3], [4], [5], [6]. Activation of caspase 3, an apoptotic effector protease involved in cleavage of tau [7] and amyloid precursor protein [8], has been found in GVDs, but rarely in other pathological structures [9], [10], [11], [12]. The protein kinases glycogen-synthase kinase 3 and casein kinase 1, which phosphorylate tau, are also markers of GVD [13], [14], [15], [16], [17]. Phosphorylated pancreatic endoplasmic reticulum kinase, a marker of a cellular stress response to unfolded protein, which is increased in AD, is associated with GVD [18]. Intraneuronal dot-like structures morphologically similar to GVDs were also labelled by phosphorylation-dependent TAR DNA binding protein (TDP43) antibody [19], in line with the abnormal TDP43 immunoreactivity reported in AD [20], [21], [22], [23], [24], [25]. Furthermore, both proteasome and endosome pathway dysfunction may be present in GVD-containing cells, as GVD has been detected by antibodies to a cellular marker of proteasome degradation, ubiquitin (Ub) [2], [26], to intermediaries in the ubiquitin system, phospho-β-catenin [27] and Pin1 [28], and to the endosome-related protein charged multivesicular body protein 2b (CHMP2B) [29], [30]. In relation to other pathognomonic features, several lines of evidence have suggested that GVDs appear within the hippocampal pyramidal neurons in AD when phosphorylated tau begins to aggregate into early-stage neurofibrillary tangles (NFTs) [11], [15], [18], [31].

However, GVDs are not AD-specific hallmark: they have been reported within the hippocampal pyramidal neurons in normal aged brain [32], as well as in other diseases such as progressive supranuclear palsy (PSP) [33], pantothenate kinase-associated neurodegeneration (PKAN) [34], corticobasal degeneration (CBD) [35] and Pick's disease (PiD) [36]. Given that all these disorders can present with pathological lesions containing phosphorylated tau protein, these findings raise the possibility that GVDs may also appear in relation to the hippocampal phosphorylated tau accumulation in non-AD neurodegenerative disorders.

Recently, we have shown that an antibody to CHMP2B can specifically detect GVDs within hippocampal pyramidal neurons in AD [29]. The high sensitivity and specificity of this antibody were later confirmed by another group [30]. CHMP2B is a component of the endosomal sorting complex required for transport III (ESCRT-III), which is involved in endocytic trafficking of proteins [37]. ESCRT-III drives the formation and specifically the scission of intraluminal vesicles in multivesicular bodies, and under certain conditions remains associated with them.

To better understand GVD formation, particularly focusing on its relationship with the accumulation of phosphorylated tau, we examined GVDs in non-AD neurodegenerative disorders. The aims of the present study were: (1) to compare the CHMP2B immunopositivity of the hippocampal GVDs in several neurodegenerative disorders that can present with pathological lesions containing phosphorylated tau protein; and (2) to investigate the association of CHMP2B-positive GVDs with tau pathology, to determine whether CHMP2B-positive GVDs and phosphorylated tau coexist among non-AD neurodegenerative disorders.

## Results

### CHMP2B-positive granules correspond to GVDs in the hippocampal neurons from patients with several neurodegenerative disorders

Immunohistochemical localization of CHMP2B was investigated in the hippocampus of several neurodegenerative disorders, including MyD, ALS-D, PDD, MSA, PiD, PSP and PKAN cases. As reported, CHMP2B immunoreactivity was observed as granules in pyramidal neurons. No immunoreactivity was detected in glial cells. CHMP2B-positive granules were often surrounded by a clear halo and were morphologically similar to the classic granules of GVD.

**Figure 1:**
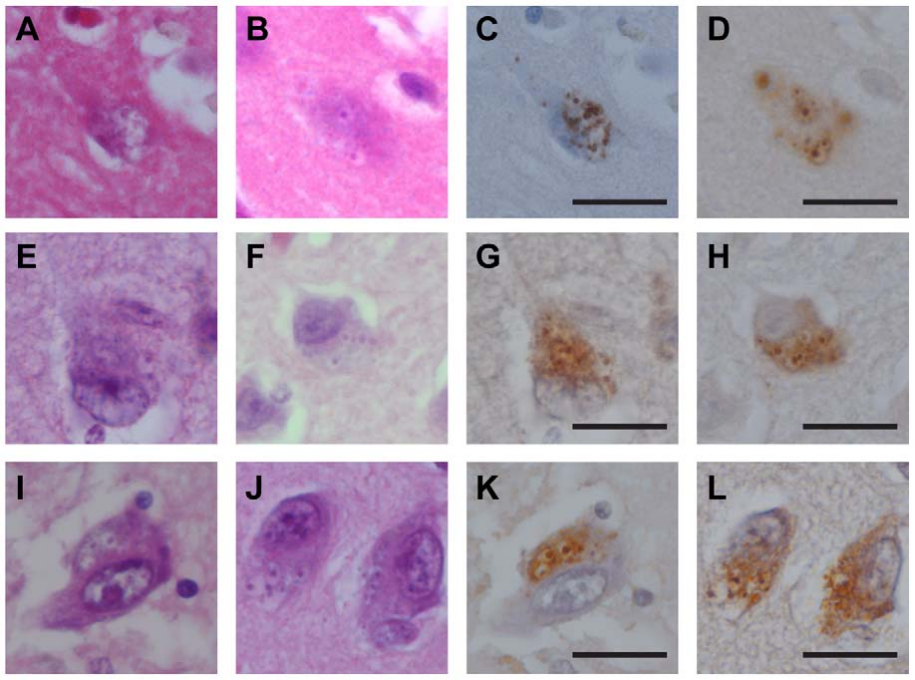
CHMP2B-positive granules correspond to GVDs. Cellular localization of CHMP2B (**C, D, G, H, K, L**) compared with hematoxylin and eosin (HE) staining (**A, B, E, F, I, J**) in several neurodegenerative disorders is shown. CHMP2B-positive structures colocalized with the GVDs identified by HE staining and surrounded by a clear halo. **A, C**, Alzheimer's disease; **B, D**, myotonic dystrophy; **E, G**, amyotrophic lateral sclerosis with dementia; **F, H**, Pick's disease; **I, K**, multiple system atrophy with parkinsonism; **J, L**, pantothenate kinase-associated neurodegeneration. Scale bars represent 20 µm.

To confirm the GVD nature of these CHMP2B-positive granules, sections were stained once with hematoxylin and eosin (Fig. 1A, B, E, F, I, J). After observing GVDs in the hippocampus, these stained sections were de-stained in absolute ethanol, and processed for CHMP2B immunohistochemical analysis (Fig. 1C, D, G, H, K, L). Most neurons with GVDs showed CHMP2B-positive granules and these CHMP2B-positive granules corresponded to GVDs. Together with our previous results, this suggested that CHMP2B could be used as a molecular label to study GVD in non-AD neurodegenerative disorders. The numbers of neurons with CHMP2B-positive GVDs/mm^2^ in each case are listed in Table S1.

### CHMP2B-positive GVDs colocalize with pSmad2/3 and ubiquitin

**Figure 2:**
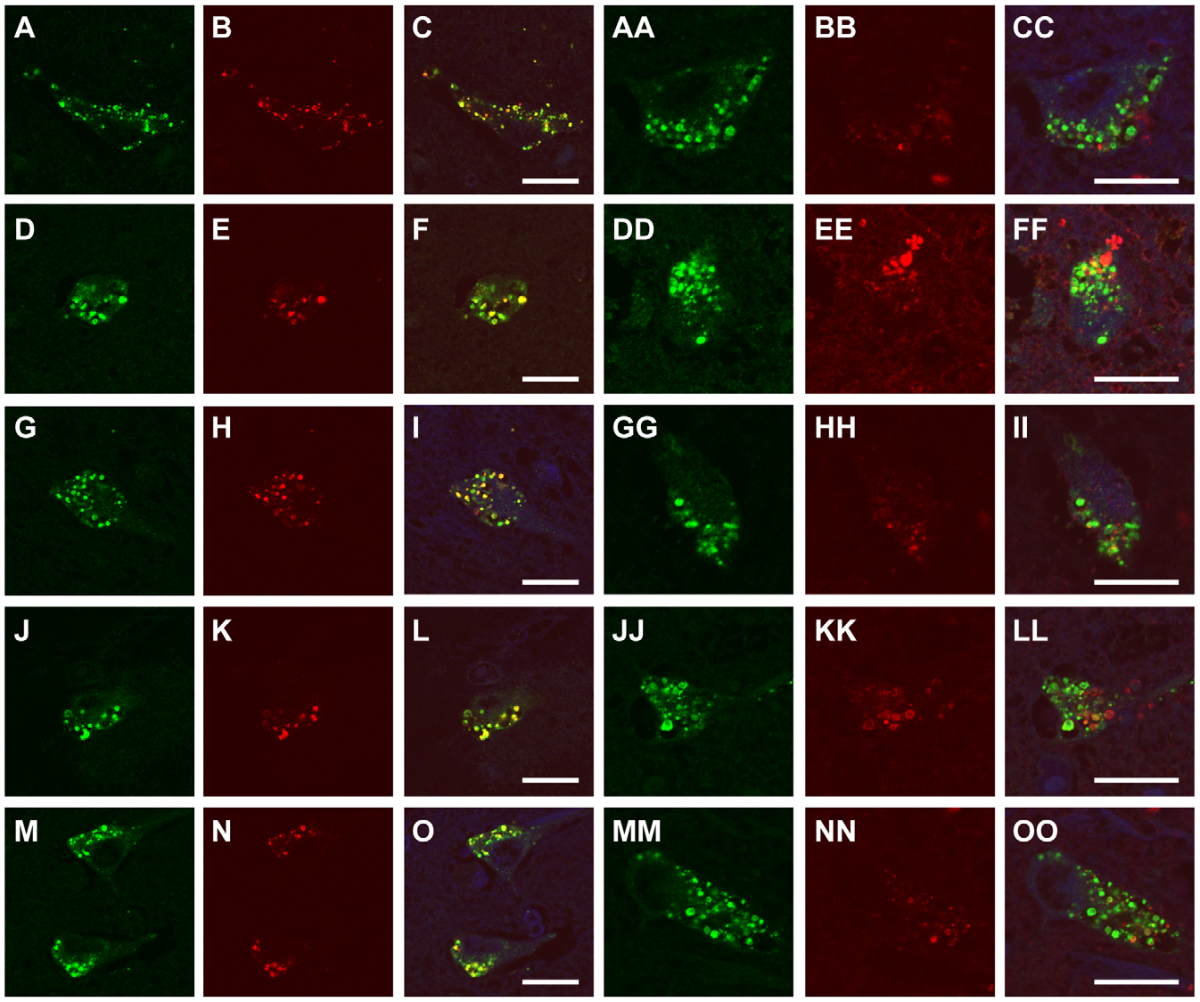
CHMP2B-positive GVDs colocalize with pSmad2/3 and ubiquitin. Hippocampal sections were stained with both anti-CHMP2B antibody (**A, D, G, J, M, AA, DD, GG, JJ, MM**, *green*) and antibodies against pSmad2/3(**B, E, H, K, N**, *red*) or ubiquitin (**BB, EE, HH, KK,** NN, *red*). CHMP2B-positive GVDs colocalized with pSmad2/3 and ubiquitin, although their colocalization rates varied. **C, F, I, L, O, CC, FF, II, LL, OO**, merged images counterstained with Hoechst Dye (*blue*). **A, B, C, AA, BB, CC**, amyotrophic lateral sclerosis; **D, E, F, DD, EE, FF**, pantothenate kinase-associated neurodegeneration; **G, H, I, GG, HH, II**, multiple system atrophy with cerebellar ataxia; **J, K, L, JJ, KK, LL**, progressive supranuclear palsy; **M, N, O, MM, NN, OO**, Parkinson disease with dementia. Scale bars represent 20 µm.

Since GVDs have been reported to be immunoreactive for pSmad2/3 [38] and Ub [2], [26], we assessed the colocalization of CHMP2B-positive GVDs and these markers using double immunofluorescent labeling. In the hippocampus of several neurodegenerative disorders, including MyD, ALS-D, PDD, MSA, and PKAN cases, almost all CHMP2B-positive GVDs were also immunopositive for pSmad2/3 and Ub (Fig. 2). The colocalization of CHMP2B-positive GVDs and these markers could also be observed even in PSP (Fig. 2J, K, L, JJ, KK, LL) and MSA-C cases (Fig. 2G, H, I, GG, HH, II), in which hematoxylin and eosin staining revealed relatively few GVDs that we could not confirm the GVD nature of CHMP2B-positive granules.

**Figure 3:**
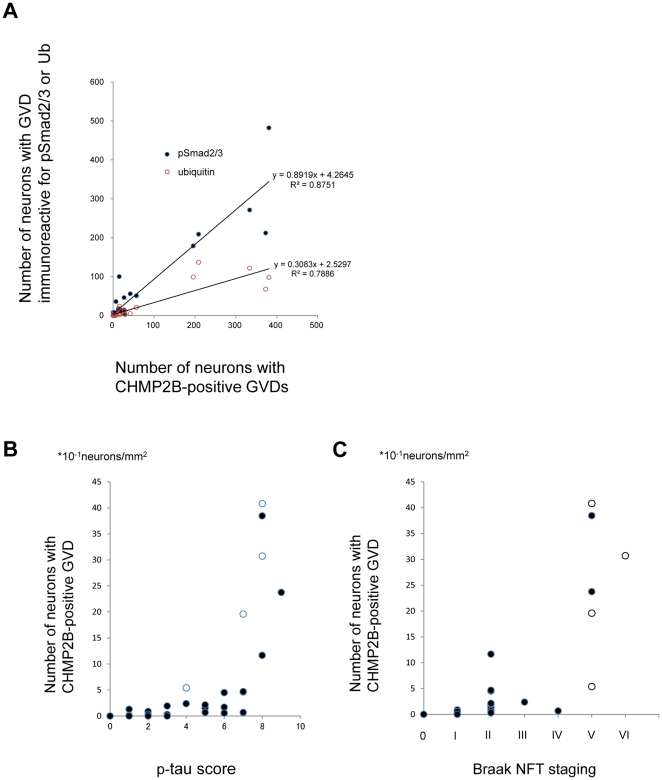
Correlation between the numbers of CHMP2B-positive GVDs with numbers of GVDs immunoreactive for pSmad2/3 or Ub and hippocampal tau pathology. **A:** Number of neurons with CHMP2B-positive GVDs plotted against those with GVDs immunoreactive for pSmad2/3 (*blue filled circles*) or ubiquitin (Ub; *red open circles*). The Pearson correlation coefficient for pSmad2/3 and ubiquitin was 0.935 (*p*<0.01) and 0.888 (*p*<0.01), respectively, among all the cases studied. Each circle represents an individual hippocampus investigated. **B:** Relationship between phosphorylated tau score (p-tau score) and the number of neurons with CHMP2B-positive GVDs in non-Alzheimer's disease (AD) neurodegenerative disorders. The number of neurons with CHMP2B-positive GVDs increased significantly with the score for phosphorylated tau pathology (r = 0.60, *p*<0.01). Each *closed circle* represents an individual non-AD hippocampus investigated. Each *open circle* represents an individual hippocampus from an AD case, for reference. **C:** CHMP2B-positive GVD burden in the hippocampus with respect to Braak NFT stage in non-Alzheimer's disease (AD) neurodegenerative disorders. The number of neurons with CHMP2B-positive GVDs increased significantly with respect to Braak NFT stage (r = 0.73, *p*<0.01). Each *closed circle* represents an individual non-AD hippocampus investigated. Each *open circle* represents an individual hippocampus from an AD case for reference.

To investigate the reliability of using CHMP2B as a molecular label to study GVDs, we next examined the correlation between the number of neurons with CHMP2B-positive GVDs and the number of neurons with granules immunopositive for pSmad2/3 or Ub. As shown in Figure 3A, immunohistochemistry with the anti-CHMP2B antibody detected GVDs in a similar number of cells to those immunoreactive for pSmad2/3 or Ub within the hippocampal region in each of the diseases studied.

### CHMP2B-positive GVDs correlate with hippocampal tau pathology phosphorylated at Ser-202 and Thr-205

Our research interest was the association of CHMP2B-positive GVDs with tau pathology in the hippocampus, including the subiculum, CA2 and CA1 subfields, where GVDs were found in high number in AD as well as non-AD cases [18], [39]. Therefore we directly compared the number of hippocampal neurons with CHMP2B-positive GVDs with the number of neurons positive for phosphorylated tau. The number of neurons positive for phosphorylated tau was assessed using our method for scoring tangle densities (for details see the Material and Methods). We also investigated the association between neurons with CHMP2B-positive GVDs and classic Braak NFT stage. The phosphorylated tau score (p-tau score) and Braak stage score in each case are listed in Table 1, right column.

**Table 1:** Scoring system used to quantify neurons with phosphorylated tau.

Structures per field	phosphorylated tau score (p-tau score)
0	0
1–2	1
3–4	2
5–9	3
10–14	4
15–19	5
20–24	6
25–29	7
30–34	8
35–40	9
> 40	10

When age at death was controlled, strong correlations were observed between both the number of neurons with CHMP2B-positive GVDs and p-tau score (r = 0.63, p<0.01) as well as CHMP2B-positve GVDs and Braak NFT stage (r = 0.77, p<0.01) across the entire sample. Strong correlations were also observed when we excluded AD cases from the analysis (r = 0.60, p<0.01, Fig. 3B, r = 0.73, p<0.01, Fig. 3C, respectively).

**Figure 4:**
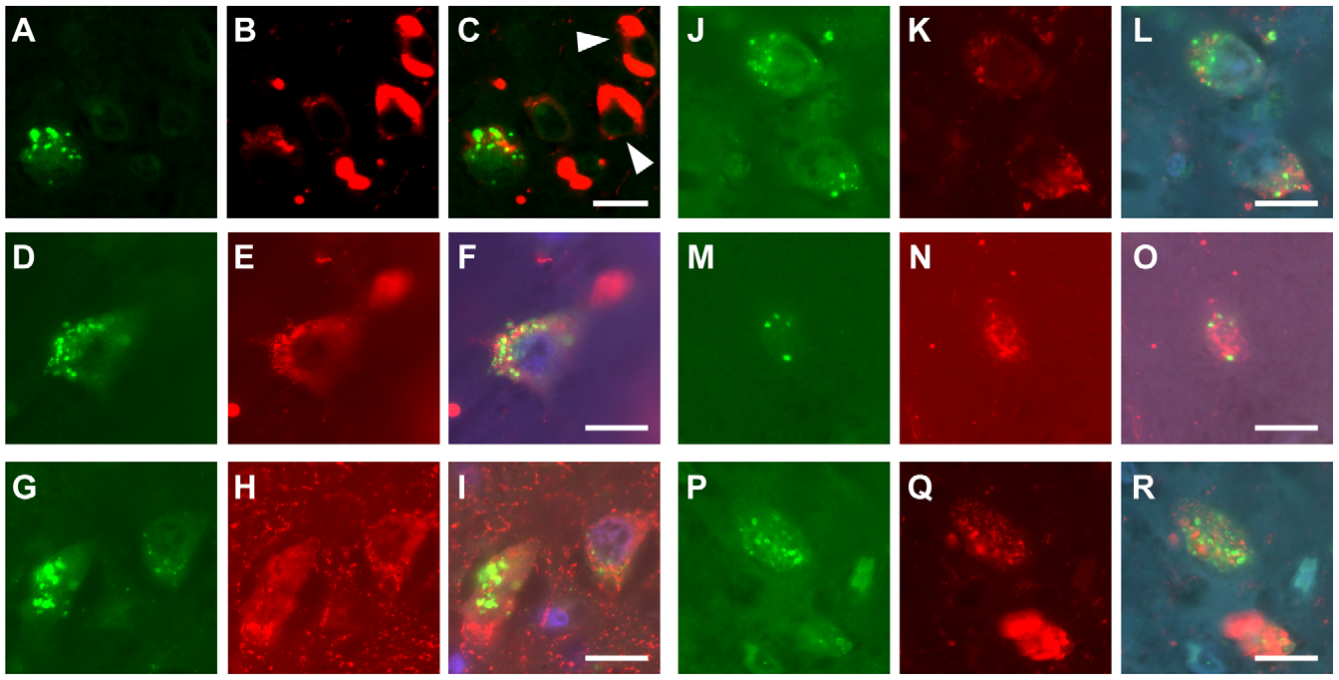
Correlation between CHMP2B-positive GVDs and phosphorylated tau at the cellular level. Double immunofluorescence labeling for CHMP2B (**A, D, G, J, M, P**; *green*), tau phosphorylated at Ser-202 and Thr-205 (**B, E, H, K, N, Q**; *red*), and merged images (**C, F, I, L, O, R**) in sections from patients with several neurodegenerative disorders is shown. Note that, in neurons containing CHMP2B-positive GVDs, diffuse staining for phosphorylated tau could be detected in Alzheimer's disease. In contrast, no co-occurrence of CHMP2B-positive GVDs and phosphorylated tau in tangle-bearing neurons (**arrowheads**) was observed in Alzheimer's disease (**A, B, C**). A similar co-occurrence of CHMP2B-positive GVDs and diffuse staining for phosphorylated tau in neurons was observed in several other neurodegenerative disorders (**D–R**). **D, E, F**, myotonic dystrophy; **G, H, I**, multiple system atrophy with parkinsonism; **J, K, L**, progressive supranuclear palsy; **M, N, O**, Parkinson disease with dementia; **P, Q, R**, pantothenate kinase-associated neurodegeneration. Merged images were counterstained with Hoechst Dye (**F, I, L, O, R**; *blue*). Scale bars represent 20 µm.

We next investigated the correlation between CHMP2B-positive GVDs and phosphorylated tau at the cellular level. For immunohistochemistry, we used the AT8 antibody, which recognizes tau phosphorylated at Ser-202 and Thr-205. In addition to NFTs, in AD and other neurodegenerative diseases AT8 stains some non-tangle-bearing pyramidal neurons, indicative of hyperphosphorylated tau in a pre-tangle stage [40]. While no or very few CHMP2B-positive GVDs were observed in neurons with NFTs immunoreactive for AT8, diffuse staining for phosphorylated tau was observed in neurons with CHMP2B-positive GVDs in several neurodegenerative disorders, except for the cases in which immunohistochemistry revealed relatively few CHMP2B-immunoreactive neurons (Table S1 and Fig. 4). Together, these data indicate that CHMP2B-positive GVDs appear in association with the accumulation of phosphorylated tau in several neurodegenerative disorders.

## Discussion

In this study, we demonstrated that CHMP2B-positive granules corresponded to GVDs in a variety of neurodegenerative disorders. The GVD nature of these CHMP2B-positive granules was demonstrated on morphological grounds and because of the strong co-localization upon both HE staining and with other GVD markers including pSmad2/3 [38] and Ub [2], [26]. In addition, the number of CHMP2B-positive GVDs was comparable with that of neurons with granules immunopositive for the GVD markers (pSmad2/3 and Ub) among the different diseases. Indeed, while GVDs are often assumed to be a pathological entity associated with AD, they have been described within the hippocampus in a number of neurodegenerative diseases; in a preceding investigation, other GVD markers including CK1 delta [17] and p-SAPK/JNK [41] also confirmed the presence of GVDs in the brains of patients with non-AD neurodegenerative disorders. Compared with p-SAPK/JNK, with which immunohistochemistry revealed pathological accumulations of NFTs in addition to GVDs [41], the advantage of CHMP2B as well as CK1 delta as a GVD marker is that it specifically stains GVDs but no other coexisting structures. Taken together, it is reasonable to argue that along with CK1 delta, CHMP2B is a robust marker of GVD in that it specifically detected GVD in AD as well as in non-AD neurodegenerative disorders.

In AD, several lines of evidence have suggested that GVDs appear within the hippocampal pyramidal neurons when phosphorylated tau begins to aggregate into early-stage NFTs [11], [15], [18], [31]; however, whether one can adapt this relationship to non-AD diseases had not yet been systematically examined [42]. In this study, we showed that the number of neurons with CHMP2B-positive GVDs increased in association with phosphorylated tau accumulation in the hippocampus not only in AD but also in a wide range of non-AD neurodegenerative disorders. In addition, we provided direct evidence that diffuse staining for phosphorylated tau could be detected in neurons with CHMP2B-positive GVDs in most of the non-AD cases including PSP and PDD. The pathological forms of tau from AD and PDD patients demonstrate four bands on western blots (72, 68, 64, and 60 kDa; Type I pattern) [43], [44], while pathological tau from PSP [45] demonstrates three bands (72, 68, and 64 kDa; Type III pattern). Unfortunately, we could not show colocalization of CHMP2B-positive GVDs with phosphorylated tau in PiD, which demonstrates two bands (64 and 60 kDa; Type II pattern) on western blots [46], [47], probably because of the small number of neurons with CHMP2B-positive GVDs. Therefore, our results suggested that in most hippocampal neurons harboring GVDs, they appear in relation to phosphorylated tau accumulation in non-AD neurodegenerative disorders including the ‘tauopathies’. However, further studies are needed to clarify whether GVDs appear in relation to phosphorylated tau in tauopathies, irrespective of the phosphorylated tau isoforms.

The most notable finding of the present study is the presence of GVDs in the phosphorylated tau-containing neurons in various neurodegenerative disorders other than AD. Although this finding raises the possibility that there is a common mechanism for GVD formation and phosphorylated tau accumulation, the cellular fates of GVD-harboring neurons may differ between AD and non-AD disorders. Previous studies have suggested that neurons harboring GVDs with phosphorylated tau accumulation reflected ‘toxic’ or ‘apoptotic’ alterations in AD [11], [18], based on their relationship with phosphorylated tau, whose degree of accumulation correlates with neuronal loss in the hippocampus [42], [48], [49], as well as the hippocampal vulnerability, both of which have been extensively characterized and documented in AD [50]. Moreover, an exponential relationship exists between the number of GVDs and neuronal loss observed in AD [42]. In contrast, a correlation between tau accumulation and cognitive decline or neuronal loss does not necessarily exist in other disorders [51], [52], and it is not known whether the same exponential relationship applies to non-AD cases [42]. Therefore, it is unclear whether hippocampal neurons harboring GVDs with tau accumulation observed in non-AD disorders are pathognomonic for phosphorylated tau-related neurodegeneration, or perhaps are an underlying toxic moiety.

It is unclear whether the presence of phosphorylated tau in the hippocampus of non-AD cases is necessarily more than a result of normal aging. Tau proteins can become insoluble with aging and sometimes contaminate the preparations of the so-called pathological tau aggregates [53]–indeed this contamination has been described in several neurodegenerative diseases [54], [55], [56]. Moreover, the NFT burden to discriminate between the aging and AD has been thought to be quantitative rather than qualitative [57], [58]. Therefore, given that our study lacks age-matched controls, we are uncertain whether the presence of phosphorylated tau in the hippocampus of non-AD cases reflects aging, a substantial AD process, or a disease-specific finding. Nonetheless, strong correlations were observed in our cases between the number of neurons with CHMP2B-positive GVDs and phosphorylated tau burden, even when we excluded AD cases from the analysis. This suggests that, whatever the mechanism involved in phosphorylated tau accumulation, GVDs appear in relation to hippocampal phosphorylated tau accumulation in various neurodegenerative disorders.

### Conclusions

In summary, using an antibody to CHMP2B as a molecular label for GVDs [29], we have shown that GVDs appear consistently with hippocampal phosphorylated tau accumulation in various neurodegenerative disorders. Although GVDs in non-AD neurodegenerative disorders have not been studied thoroughly, our results suggested that they are not incidental findings but rather they appear in relation to phosphorylated tau accumulation, further highlighting the role of GVD in the process of phosphorylated tau accumulation.

## Materials and Methods

### Ethics Statement

The protocols for neuropathological procedures and analysis were approved by and performed under the guidelines of the ethics committee of Hiroshima University Graduate School of Biomedical Sciences. The neurodegenerative disorders and control samples were obtained with the adequate understanding and written informed consent of family members. For this study, all samples were coded and personal information dissociated from the test results. All the data were analyzed anonymously, and all neuropathological procedures and analysis have been conducted according to the principles expressed in the Declaration of Helsinki.

### Brain pathology and staining

Four cases of AD, five cases of myotonic dystrophy (MyD), eight cases of amyotrophic lateral sclerosis, two cases of ALS with dementia (ALS-D), three cases of Parkinson disease with dementia (PDD), and one case each of multiple system atrophy with parkinsonism, multiple system atrophy with cerebellar ataxia (MSA-C), PiD, PSP, CBD, and PKAN, and nine control cases without neurodegenerative disorders according to clinical history and confirmed by thorough neuropathological examination were selected (for case demographics see Table S1, postmortem delays 4–24 hours).

Formalin-fixed, paraffin-embedded tissues including the hippocampus and the parahippocampal gyrus were sliced at a thickness of 7 µm. The sections were deparaffinized and then immunostained with primary antibody. The primary antibodies used were as follows: rabbit polyclonal antibody to CHMP2B (ab33174, dilution 1∶600; Abcam, Cambridge, UK); mouse monoclonal antibody to ubiquitin (MAB1510, dilution 1∶2,000; Chemicon, Temecula, CA); goat polyclonal antibody to pSmad2/3 (sc-11769, dilution 1∶400; Santa Cruz Biotech, Santa Cruz, CA); and mouse monoclonal antibody to phosphorylated tau (AT8, dilution 1∶800; Innogenetics, Gent, Belgium). For antigen retrieval, the slides were microwaved in distilled water for 10 min then washed in phosphate-buffered saline (PBS) for 5 min. Deparaffinized sections were then incubated with 1% H_2_O_2_ in methanol for 20 min to eliminate endogenous peroxidase activity. Each section was incubated with primary antibody overnight at 4°C. After washing in PBS, the sections were incubated with horseradish peroxidase (HRP)-conjugated goat anti-mouse antibody or goat anti-rabbit antibody (both diluted 1∶100; DAKO, Glostrup, Denmark) for 30 min at room temperature. The sections were then washed three times in PBS and incubated at room temperature with 3,3′-diaminobenzidine (DAKO). All sections were counterstained with hematoxylin.

In addition, we performed double staining on sections including those of the hippocampus and the parahippocampal gyrus of the disease cases. We applied the same primary antibodies as described above. These primary antibodies were detected with the following secondary antibodies (dilution 1∶500; Molecular Probes, Eugene, OR): Alexa Fluor 488 donkey anti-rabbit IgG, Alexa Fluor 546 donkey anti-mouse IgG, Alexa Fluor 546 donkey anti-goat IgG, Alexa Fluor 488 goat anti-mouse IgG, and Alexa Fluor 546 goat anti-mouse IgG. 0.5% Sudan black in 70% ethanol was used to quench autofluorescence before mounting the paraffin-embedded sections. The slides were mounted with Vectashield (Vector Laboratories, Burlingame, CA), and observed under a fluorescence microscope (BIOREVO BZ-9000; Keyence, Osaka, Japan) or an LSM510 confocal laser scanning microscope (Carl Zeiss AG, Oberkochen, Germany).

We assessed the staining specificity by replacing the primary antibodies with an appropriate amount of non-immune rabbit serum or PBS containing 3% bovine serum albumin or by pre-incubating the primary antibodies with an excess of peptide immunogen. No reaction products were seen in the sections thus treated (data not shown).

### Quantitative analysis

For all cases, we counted the number of neurons with CHMP2B-positive GVDs and neurons with granules immunopositive for pSmad2/3 and Ub in the hippocampus at 400× magnification. For the analysis of the correlation to the tau pathology, the number of neurons with CHMP2B-positive GVDs was expressed as neurons/mm^2^; we used a mean number of three independent measures using light microscopy at 400× magnification and the images of total hippocampal area in each case were measured by the Image J (NIH) software.

The accumulation of phosphorylated tau in the hippocampus was evaluated using the grading score of Mölsä with some modifications [59]. Briefly, AT8-stained sections including the hippocampus were scanned using light microscopy at 100× magnification. Five randomly selected fields, each measuring 0.92 mm^2^, were selected, and the mean number of neurons positive for phosphorylated tau in each field was calculated (Table 1). A lesion score was then assigned, ranging from 0 to 10. For example, if the lowest neurons with phosphorylated tau density were 10–14 and the highest 26–30, the range would be 10–30, giving a midpoint of 20 and phosphorylated tau score (p-tau score) of 6. If the highest density were over 40, the arbitrary figure of 45 was used when calculating the midpoint. Braak staging was determined by AT8 immunostaining of neurofibrillary tangles in hippocampus and isocortical brain regions [60].

Statistical evaluations were performed with the SPSS 14 software package (SPSS, Chicago, Illinois). Due to the exploratory nature of our investigation, the level of significance was set to .05 (two-tailed tests). To avoid effects of aging, we used partial correlations to analyze the relations between number of neurons with CHMP2B-positive GVDs and phosphorylated tau accumulation. Partial correlation analysis reduces the potential for misleading interpretation of data and in the current study, provided a more rigorous investigation of the relationships between the variables. We interpreted coefficients >.5 as strong correlations [61].

## Supporting Information

Table S1 Description of cases studied.
(DOC)
