# Supplementary material for: Granulovacuolar Degenerations Appear in Relation to Hippocampal Phosphorylated Tau Accumulation in Various Neurodegenerative Disorders
Source: PLoS One. 2011 Nov 3;6(11):e26996. doi: 10.1371/journal.pone.0026996 (PMC3207829; doi:10.1371/journal.pone.0026996)
Supplement: Table S1 — Description of cases studied. (DOC) [file pone.0026996.s001.doc]

**Table S1**

Description of cases studied.

| Case | Age (years) | Sex | Number of hippocampal neurons with CHMP2B-positive GVDs (*10^-1^neurons/mm^2^) | Rank of tau pathology  (phosphorylated tau score) | Braak stage | Correlation between CHMP2B-positive GVD and phosphorylated tau at the cellular level |
| --- | --- | --- | --- | --- | --- | --- |
| AD 1 | 85 | F | 40.8 | 8 | 5 | confirmed |
| AD 2 | 72 | F | 5.4 | 4 | 5 | confirmed |
| AD 3 | 75 | F | 30.7 | 8 | 6 | confirmed |
| AD 4 | 66 | F | 19.6 | 7 | 5 | confirmed |
| MyD 1 | 59 | F | 1.7 | 6 | 2 | confirmed |
| MyD 2 | 57 | M | 4.6 | 7 | 2 | confirmed |
| MyD 3 | 59 | M | 2.1 | 5 | 2 | confirmed |
| MyD 4 | 63 | M | 1.5 | 5 | 2 | confirmed |
| MyD 5 | 48 | M | 0.0 | 1 | 1 | not examined |
| ALS-D 1 | 74 | F | 38.5 | 8 | 5 | confirmed |
| ALS-D 2 | 74 | F | 1.3 | 1 | 2 | confirmed |
| ALS 1 | 75 | M | 0.9 | 2 | 1 | unconfirmed |
| ALS 2 | 67 | F | 0.0 | 1 | 1 | not examined |
| ALS 3 | 72 | F | 4.5 | 4 | 2 | confirmed |
| ALS 4 | 65 | M | 0.3 | 3 | 1 | unconfirmed |
| ALS 5 | 70 | F | 1.9 | 3 | 2 | confirmed |
| ALS 6 | 73 | M | 0.1 | 1 | 1 | unconfirmed |
| ALS 7 | 63 | M | 0.0 | 2 | 1 | not examined |
| ALS 8 | 76 | F | 11.7 | 8 | 2 | confirmed |
| PDD 1 | 71 | M | 0.6 | 6 | 1 | confirmed |
| PDD 2 | 75 | F | 2.4 | 4 | 3 | confirmed |
| PDD 3 | 86 | M | 4.7 | 7 | 2 | confirmed |
| MSA-P | 73 | F | 2.1 | 5 | 2 | confirmed |
| MSA-C | 72 | F | 0.7 | 7 | 2 | unconfirmed |
| PiD | 66 | M | 0.7 | 5 | 4 | unconfirmed |
| PSP | 60 | F | 0.3 | 2 | 2 | confirmed |
| CBD | 75 | F | 0.0 | 2 | 0 | not examined |
| PKAN | 57 | F | 23.8 | 9 | 5 | confirmed |
| Co 1 | 64 | F | 0.0 | 1 | 0 | not examined |
| Co 2 | 80 | M | 0.0 | 0 | 1 | not examined |
| Co 3 | 26 | F | 0.0 | 0 | 1 | not examined |
| Co 4 | 56 | M | 0.0 | 0 | 1 | not examined |
| Co 5 | 29 | F | 0.0 | 0 | 0 | not examined |
| Co 6 | 29 | M | 0.0 | 0 | 0 | not examined |
| Co 7 | 42 | F | 0.0 | 3 | 0 | not examined |
| Co 8 | 68 | M | 0.0 | 2 | 1 | not examined |
| Co 9 | 69 | M | 0.0 | 3 | 1 | not examined |

Abbreviations: GVD, granulovacuolar degeneration; NFT, neurofibrillary tangle; AD, Alzheimer’s disease; MyD, myotonic dystrophy; ALS-D, ALS with dementia; PDD, Parkinson disease with dementia; MSA-P, multiple system atrophy with parkinsonism; MSA-C, multiple system atrophy with cerebellar ataxia; PiD, Pick’s disease; PSP, progressive supranuclear palsy; CBD, corticobasal degeneration; PKAN, pantothenate kinase-associated neurodegeneration; Co, control; M, male; F, female.
